# The effects of restricted glycolysis on stem-cell like characteristics of breast cancer cells

**DOI:** 10.18632/oncotarget.25299

**Published:** 2018-05-01

**Authors:** Arindam Banerjee, Pardis Arvinrad, Matthew Darley, Stéphanie A. Laversin, Rachel Parker, Matthew J.J. Rose-Zerilli, Paul A. Townsend, Ramsey I. Cutress, Stephen A. Beers, Franchesca D. Houghton, Charles N. Birts, Jeremy P. Blaydes

**Affiliations:** ^1^ Cancer Sciences Unit, Faculty of Medicine, University of Southampton, Southampton, SO16 6YD, UK; ^2^ Centre for Human Development, Stem Cells & Regeneration, Faculty of Medicine, University of Southampton, Southampton, SO16 6YD, UK; ^3^ Antibody & Vaccine Group, Faculty of Medicine, University of Southampton, Southampton, SO16 6YD, UK; ^4^ University Hospital Southampton, Faculty of Medicine, University of Southampton, Southampton, SO16 6YD, UK; ^5^ Division of Molecular and Clinical Cancer Sciences, Manchester Cancer Research Centre, Manchester Academic Health Science Centre, University of Manchester, Manchester, M20 4QL, UK; ^6^ Institute for Life Sciences, University of Southampton, Southampton, SO17 1BJ, UK

**Keywords:** breast cancer stem cell-like cells, metabolism, single-cell mRNA-seq, chemoresistance, glycolysis

## Abstract

Altered glycolysis is a characteristic of many cancers, and can also be associated with changes in stem cell-like cancer (SCLC) cell populations. We therefore set out to directly examine the effect of glycolysis on SCLC cell phenotype, using a model where glycolysis is stably reduced by adapting the cells to a sugar source other than glucose. Restricting glycolysis using this approach consistently resulted in cells with increased oncogenic potential; including an increase in SCLC cells, proliferation in 3D matrigel, invasiveness, chemoresistance, and altered global gene expression. Tumorigenicity *in vivo* was also markedly increased. SCLC cells exhibited increased dependence upon alternate metabolic pathways. They also became c-KIT dependent, indicating that their apparent state of maturation is regulated by glycolysis. Single-cell mRNA sequencing identified altered networks of metabolic-, stem- and signaling- gene expression within SCLC-enriched populations in response to glycolytic restriction. Therefore, reduced glycolysis, which may occur in niches within tumors where glucose availability is limiting, can promote tumor aggressiveness by increasing SCLC cell populations, but can also introduce novel, potentially exploitable, vulnerabilities in SCLC cells.

## INTRODUCTION

Major obstacles to treat cancer include chemoresistance, relapse and metastasis. Stem cell-like cancer (SCLC) cells are critically involved in driving these processes [1]. An increased rate of glycolysis, despite sufficient oxygen for mitochondrial respiration (i.e. the Warburg effect) is a characteristic of many cancer cells [2]. However cancer cells can modulate their glucose utilization to overcome nutritional limitations in tumor microenvironments, and this metabolic plasticity can be important in driving metastasis and chemoresistance [3–5]. Studies on breast cancer have demonstrated clear differences between SCLC cells and the bulk tumor cell population in their utilization of, and dependence on, specific metabolic pathways [3, 6, 7]. Indeed, these differences may provide a therapeutic opportunity to selectively target the SCLC cells [6, 8]. However, some reports show that breast SCLC cells are dependent on glycolysis [8], whereas others find higher mitochondrial oxidative phosphorylation in SCLC cells [3, 6]. These contradictions are yet to be fully resolved, but represent some of the many examples of mechanistic interplay between major cellular metabolic pathways, the regulation of gene expression, and the control of stem cell maintenance [9–11].

Glucose concentrations within the blood are ∼5 mM, 2-4 fold less in normal tissue, and as low as 0.1 mM in tumor tissue [12]. It is therefore critical to understand how restricted glycolysis, as well as the increased propensity of the cancer cell to utilize glucose when it is available, impacts upon the phenotype of the cancer and SCLC cells. However, tumor cells in culture deplete glucose from the media at high rates, hence the common use in research of high (25 mM) glucose DMEM to ensure concentrations do not fall below physiological glucose concentrations of ∼5 mM. Consequently, the stable maintenance of cells in sub-physiological glucose concentrations in culture is not possible without the use of complex flow cells systems [13]. To circumvent this, we have previously exploited [14] an experimental model whereby glucose in the medium is substituted by an alternative sugar, fructose [15]. *In vivo*, the majority of dietary fructose is rapidly metabolized by the liver, and stored as triglycerides, with potentially systemic effects on metabolism if ingested in excess [16]. In the *in vitro* model, in cells which express the GLUT5 transporter such as breast cancer cells [17], fructose enters glycolysis as fructose-6-phosphate, which can be channeled into either glycolysis or the pentose phosphate pathway, as the glucose-6-phosphate isomerase reaction is reversible [15]. However cells are only able to import and retain fructose at ∼100 times reduced rates compared to glucose, and therefore culture in 10 mM fructose results in rates of glycolysis equivalent to those that would be achieved with stable extracellular glucose concentrations of ∼0.1-0.2 mM [15]. Such methods have proven powerful tools in the dissection of the role of glycolysis in other biological processes [11]. Using this model in the context of lines derived from different subtypes of breast cancer, we report here that glycolytic restriction not only promotes cellular invasion and chemoresistance, but also enriches for SCLC cell populations with distinct patterns of gene expression and responses to potential targeted therapeutic interventions.

## RESULTS

### Adaptation to conditions that restrict glycolysis promotes a malignant phenotype

**Figure 1:**
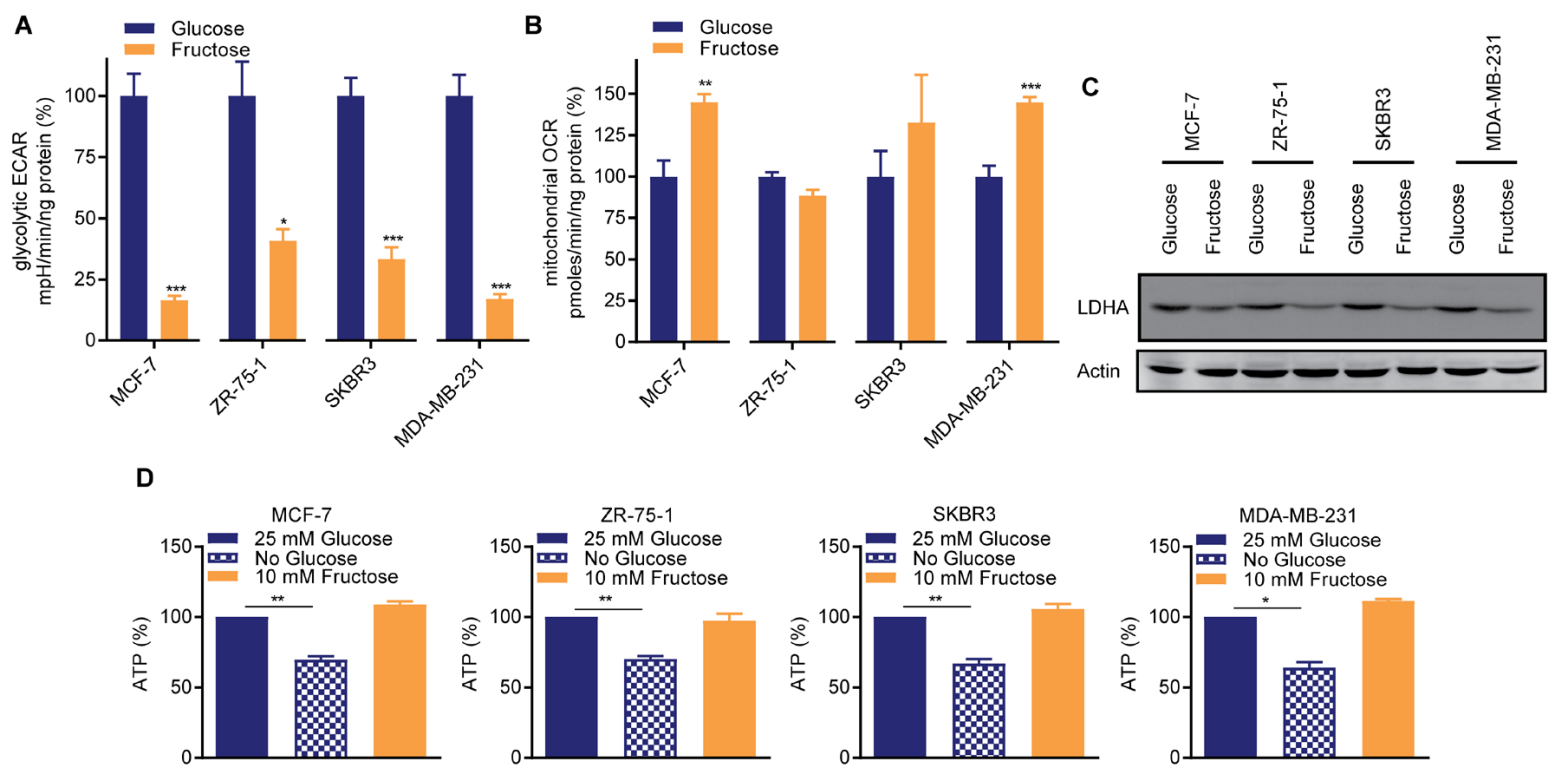
Restricted glycolysis maintains cellular bio-energetic balance in breast cancer cells Matched pairs of either 25 mM glucose- or 10 mM fructose-adapted MCF-7, ZR-75-1, SKBR3 and MDA-MB-231 cells were seeded in 2D culture conditions and **(A)** glycolytic extracellular acidification rate (ECAR) and **(B)** mitochondrial oxygen consumption rate (OCR) were analyzed by a Seahorse BioscienceXF96 Extracellular Flux Analyzer. (A, B are n=3 to 5 from a representative of ≥ 2 independent experiments. *t*-test). Values expressed relative to the glucose-cultured cells. **(C)** Western blot for lactate dehydrogenase A (LDHA). **(D)** Cellular ATP abundance assay. Glucose- and fructose-adapted cells were assayed. Glucose-cultured cells were also cultured 0 mM glucose 24 h to determine the dependence of ATP concentration on glucose metabolism in these cells. (*t*-test). In one experiment fructose-adapted cells were also similarly transferred to 0 mM fructose (Supplementary Figure 1).

Breast cancers are sub-divided into distinct subtypes based on their gene expression profiles, and cell lines representative of these subtypes have been extensively characterized [18]. Cell lines representing broad subtypes, and differentially expressing the major histological markers (MCF-7 (luminal, ER^+ve^/PR^+ve^), ZR-75-1 (luminal, ER^+ve^/PR^-ve^), SKBR3 (luminal, HER2^+ve^) and MDA-MB-231 (basal, triple negative)) were adapted to culture in otherwise identical media containing either 25 mM glucose or 10 mM fructose, as per Reitzer *et al* [15]. Consistent with this prior work [15], compared to glucose-cultured cells, fructose-adapted cells exhibited decreases in glycolysis of between 60% (ZR-75-1) and >80% (MCF-7 and MDA-MB-231) (Figure 1A). Mitochondrial oxygen consumption was either unchanged by fructose-adaptation or, in MCF-7 and MDA-MB-231, significantly increased. (Figure 1B). Expression of lactate dehydrogenase A (LDHA), which is required for high glycolytic rates [2], was decreased in the fructose-adapted cells (Figure 1C). This adaptation to glycolytic restriction did not negatively impact the ATP concentration in any of the cell lines, contrasting with the significant reduction in ATP levels when glucose-cultured cells were transiently cultured in 0 mM glucose containing medium (Figure 1D).

**Figure 2:**
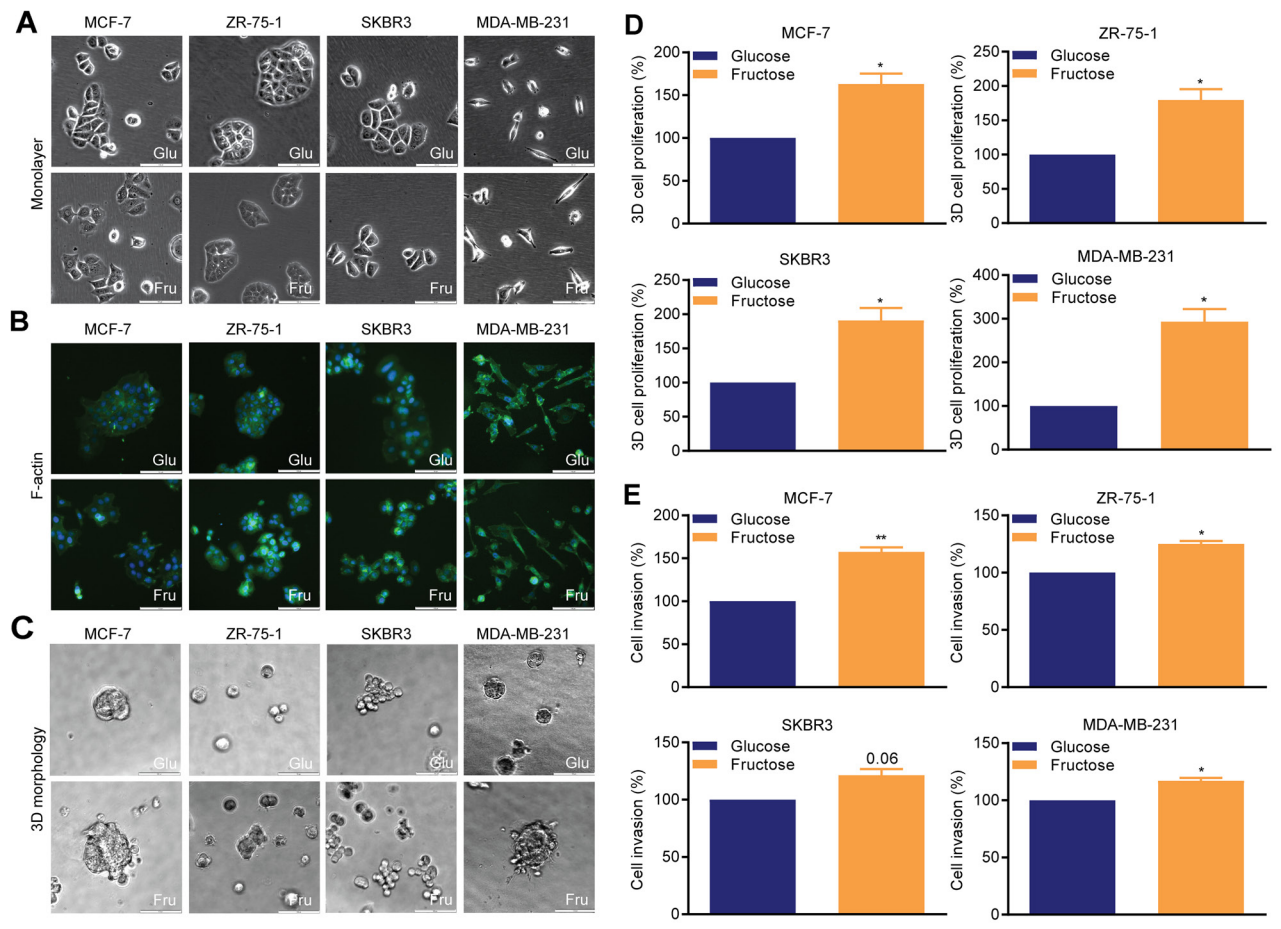
Restricted glycolysis alters cellular morphology and invasiveness in breast cancer cells **(A-C)** Cellular morphology (bar 100 μm). (A) monolayer adherent cells, (B) organization of F-actin (phalloidin-FITC), (C) 3D matrigel colonies. **(D)** 3D matrigel cell growth. **(E)** Cellinvasion assay. Mean invasionefficiency of the glucose-cultured cells was MCF-7 0.77%, ZR-75-1 1.25%, SKBR3 1.85% (all48 hour assays) and MDA-MB-231 4.2% (24 hour assays). Invasion efficiency is calculated in relation to the total number of cell plated. Representative images of invasion assays are shown in Supplementary Figure 2. (D, E) One sample *t*-test.

In the specific context of hypoxic stress, high rates of anaerobic glycolysis are associated with metastasis [19]. As the defining feature of the Warburg effect is high rates of glycolysis in the presence of oxygen, we first used our experimental model to examine the effect of glycolysis on cellular morphology, proliferation, and invasion in non-hypoxic conditions. In monolayers (Figure 2A), glucose-cultured MCF-7, ZR-75-1 and SKBR3 cells exhibited typical epithelial morphology with clear cell-to-cell adhesion, whereas MDA-MB-231 cells demonstrated a mesenchymal-like phenotype. Fructose-adapted MCF-7, ZR-75-1 and SKBR3 cells exhibited more scattered and loose colonies and MDA-MB-231 cells became more elongated. F-actin staining reinforced these observations and also identified an increase in cell membrane protrusions in fructose-adapted MCF-7 and ZR-75-1 cells (Figure 2B). In 3D matrigel (Figure 2C), glucose-cultured MCF-7, ZR-75-1 and MDA-MB-231 produced compact and circumscribed colonies, whereas fructose-adapted cells produced looser and irregular shaped colonies. Fructose-adapted SKBR3 cells generated more disorganized grape like colonies with less cell-to-cell adhesion compared to the matched glucose-cultured cells.

In all four lines, glycolytic restriction promoted cell proliferation in 3D matrigel cell cultures (Figure 2D), with the greatest effect being observed in MDA-MB-231 cells (>200% increase). Invasion through matrigel was also significantly increased in all the lines (Figure 2E), with the greatest relative effect observed in MCF-7 cells (>50% increase), though this line had the lowest initial invasive potential. MDA-MB-231 were the most invasive cell line when adapted to fructose-containing medium, with the numbers of cells invading being 5.1% of the initial cell number plated. Therefore, in contrast to the hypoxic scenario [19], under normoxia a switch to a less glycolytic phenotype can result in increased invasion in breast cancer cells.

### Stem cell-like cancer cells accumulate under conditions of restricted glycolysis

**Figure 3:**
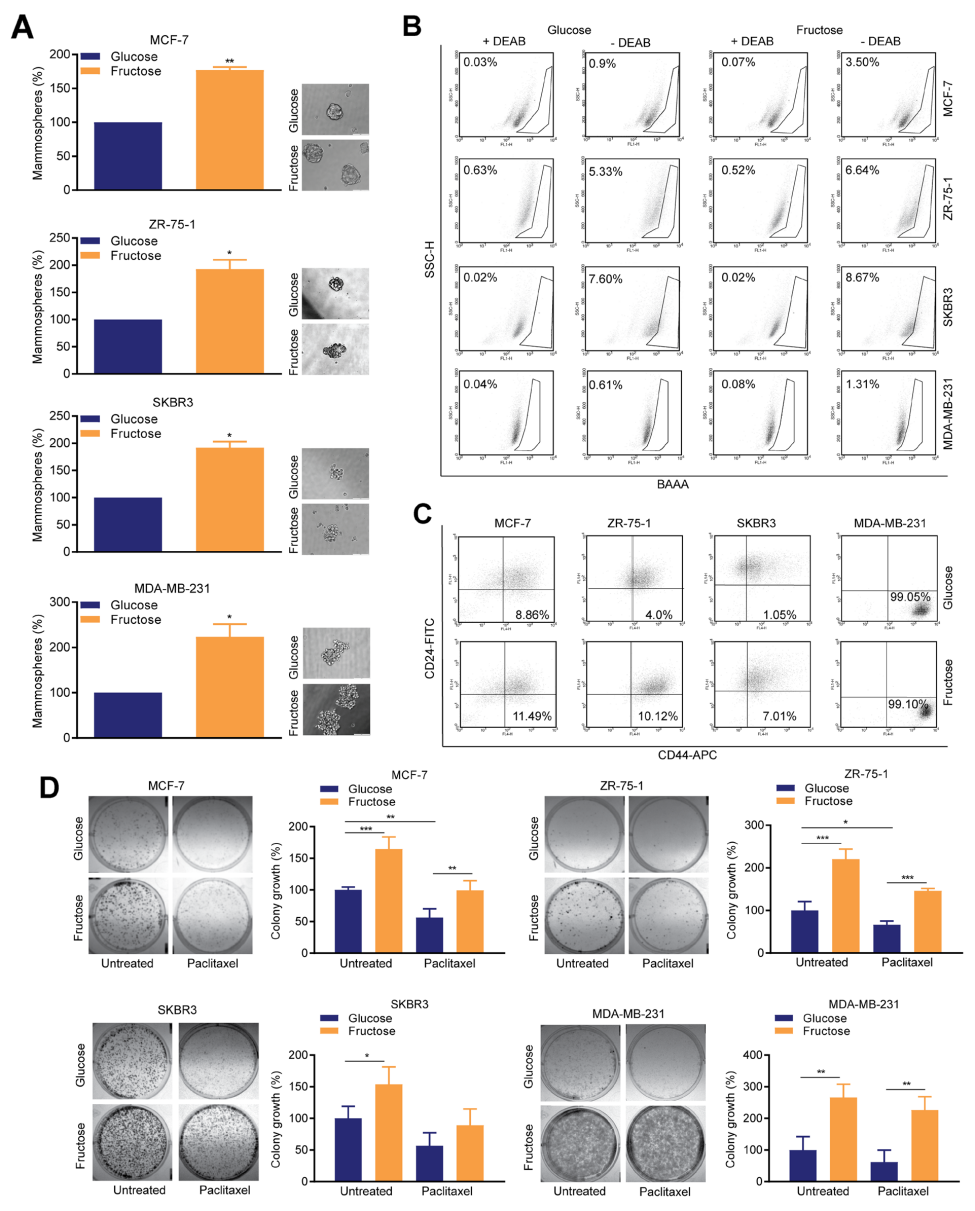
Restricted glycolysis enhances the SCLC cell population in breast cancer cells **(A)** Mammosphere assay. 8-10 days after plating in mammosphere culture conditions, representative images were captured by microscopy (bar 100 μm) and mammospheres were measured by alamarBlue. (one sample *t*-test). **(B)** ALDEFLUOR assay; diethylaminobenzaldehyde (DEAB), was used to establish the baseline fluorescence. Flow cytometry plots indicate side scatter (SSC) versus fluorescence intensity. **(C)** Flow cytometric assessment of surface CD24 and CD44 expression. (B, C) representative of one of three biological repeats. **(D)** Colony assay; 18 h after plating, media was replaced with media containing either DMSO carrier control or paclitaxel (5 nM) and cells allowed to grow for 8-10 days. (one way ANOVA and Fisher’s LSD test).

To determine how restriction of glycolysis impacts on SCLC cells, we used the mammosphere forming assay, which determines the relative numbers of SCLC cells present in the 2D cultures [20]. For all four lines, fructose-adapted cells exhibited a markedly increased ability to develop mammospheres, compared to those cultured in glucose (Figure 3A). The magnitude of this increase ranged from 77% to 123% in MCF-7 and MDA-MB-231 cells respectively. We also performed flow cytometry analysis of aldehyde dehydrogenase (ALDH) activity and surface CD44^high^/CD24^low/-ve^ status, which identify epithelial-like and mesenchymal-like SCLC cell populations, respectively [21]. Glycolytic restriction resulted in an increase in the percentage of cells with ALDH activity in all four cell lines, with the largest effect again being observed in MCF-7 and MDA-MB-231 cells (0.9% to 3.5% and 0.61% to 1.3%, respectively) (Figure 3B). The proportion of cells in the CD44^high^/CD24^low/-ve^ fraction (Figure 3C) was also increased in three of the lines, the exception being MDA-MB-231 cells which are essentially all mesenchymal-like and in which these markers are known to be uninformative with respect of SCLC cell status [22].

Increased replicative competence and reduced sensitivity to common chemotherapeutic agents are characteristic features of SCLC cells [1]. Therefore, we performed clonogenic assays with or without paclitaxel, to determine the effect of glycolytic restriction on these phenotypes (Figure 3D). For all four cell lines, fructose-adapted cells exhibited significantly greater replicative competence compared to the matched glucose pairs. The magnitude of this effect was such that, even after treatment with paclitaxel, the growth of fructose-cultured colonies was equivalent to, or greater than, the growth of untreated glucose-cultured cells. The most striking effects of fructose adaption were observed in MDA-MB-231, in which, when compared to untreated controls cultured in glucose-containing media, cells cultured in fructose showed a 166±24% increase in colony growth in the absence of paclitaxel, and still maintained a 126±24% increase when paclitaxel was added.

### Restriction of glycolysis promotes tumorigenicity in a model of triple-negative breast cancer

**Figure 4:**
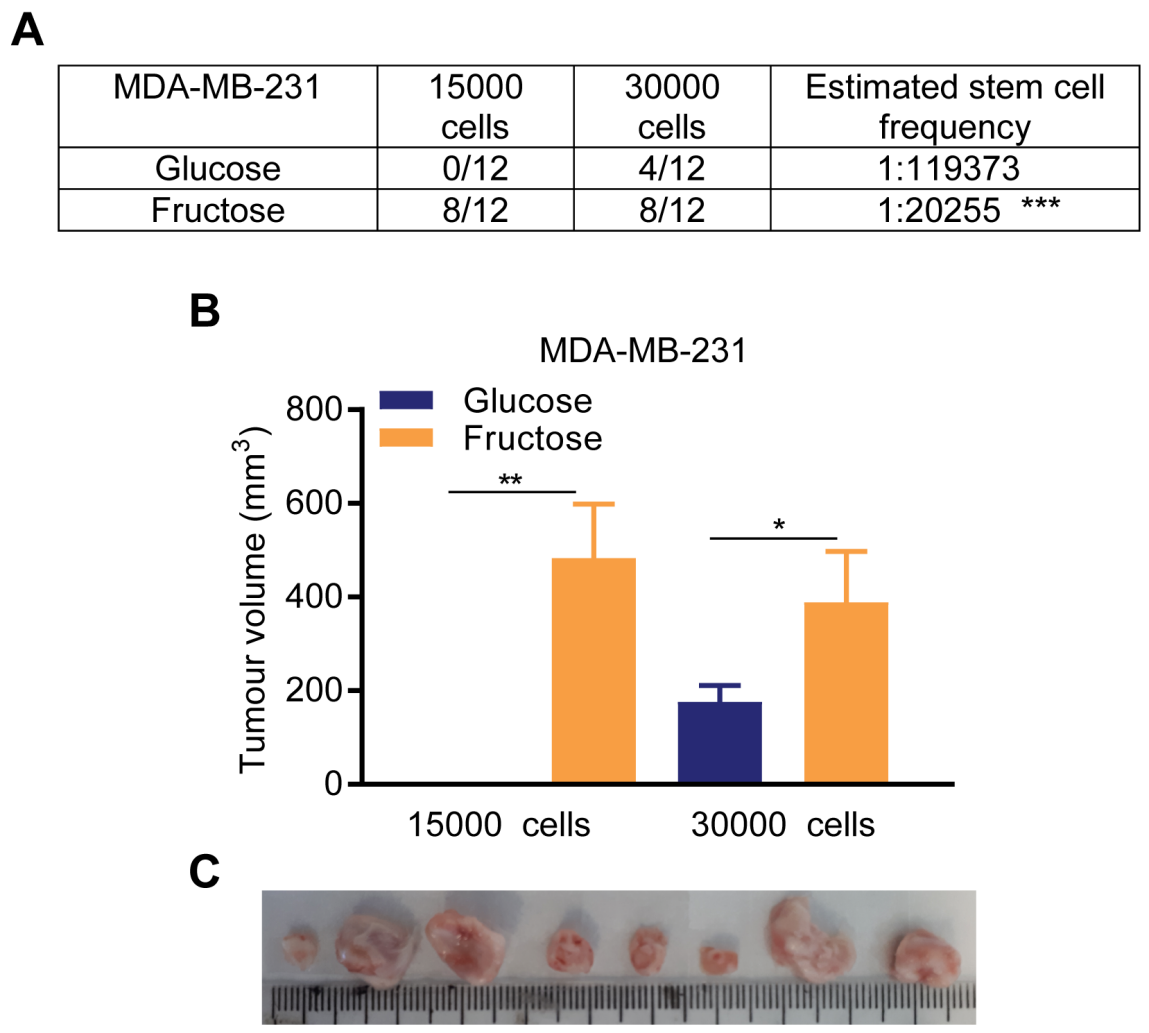
Restricted glycolysis promotes tumor initiating capacity in breast cancer cells *in vivo* **(A, B)** *In vivo* tumor initiating capacity measurement. Following optimization of the experiment model (Supplementary Figure 3), 1.5 × 10^4^ and 3 × 10^4^ cells of either glucose- or fructose-adapted MDA-MB-231 were injected on both flanks in six mice per group. (A) Counts of palpable and measurable tumors formed within 6 weeks of injection, and resultant estimation of stem cell frequency in the injected cell populations. (B) Tumor growth measurement data are presented on week 6. (Unpaired *t*-test on tumors from (A)). **(C)** Images of tumors from mice injected with 1.5 × 10^4^ cells and culled in week 6-7. All the tumors are from fructose-adapted cells. Tumors were bisected and placed with the uncut plane uppermost.

MDA-MB-231 cells form rapidly growing tumors when injected into the mammary fat pads of immunocompromised mice (Supplementary Figure 3); we therefore used these cells for *in vivo* orthotopic xenograft experiments. With 3 × 10^4^ cells injected per site, 4 out of 12 sites injected with glucose-adapted cells formed tumors (mean tumor volume 6 weeks post injection 176.4±34.6 mm^3^), whereas with fructose-adapted cells, palpable tumors formed in 8 out of 12 injection sites (388.9±108.2 mm^3^) (Figure 4). Most strikingly, when 1.5 × 10^4^ cells were injected no palpable tumors were formed from glucose-adapted cells (12 sites) whereas, with fructose-adapted cells, palpable tumors were readily detectable in 8 out of 12 injection sites in this timeframe (483.1 ±115.5 mm^3^), (Figure 4). Together, these *in vivo* data demonstrate a significant, ∼6-fold, increase in stem cell frequency in MDA-MB-231 cells when adapted to fructose, compared to culture in glucose-containing media, providing further evidence for a significant increase in SCLC cells in response to restriction of glycolysis.

### Restriction of glycolysis alters the phenotype of SCLC cells

**Figure 5:**
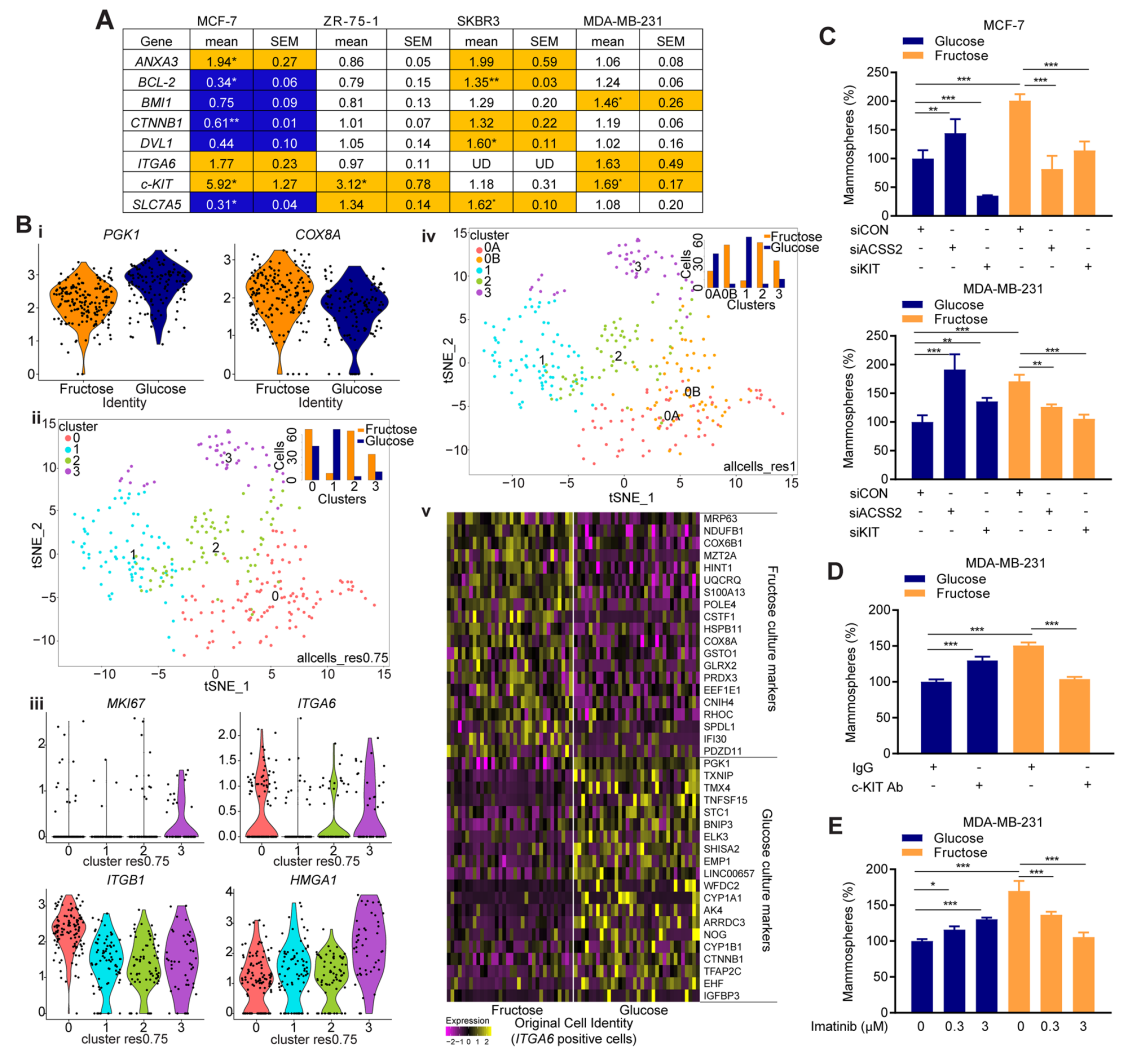
Restricted glycolysis alters metabolic signatures in breast SCLC cells **(A)** Expression of genes relevant to SCLC cell characteristics. mRNA expression in fructose-cultured cells is shown relative to the expression in glucose-cultured cells. (orange-increased in fructose, blue-increased in glucose. ≥ 25% change, UD = below limits of detection). n=3 biological repeats. **(B)** MDA-MB-231 cultured as mammospheres in either fructose or glucose containing medium were analyzed by Drop-Seq. (i) violin plots showing the effects of culture condition of the expression of *PGK1* (glycolytic enzyme) and *COX8A* (mitochondrial electron transport chain enzyme), (ii) t-SNE analysis after regression of cell cycle signatures, with cell clusters identified at resolution 0.75. Insert is a bar chart showing the number of cells from each culture condition in the clusters, (iii) violin plots of expression of specific genes in the cells in each cluster, (iv) t-SNE analysis after regression of cell cycle signatures, with cell clusters identified at resolution 1.0. Insert is a bar chart showing the number of cells from each culture condition in the clusters, (v) Heatmap of differential gene expression in glucose- versus fructose-cultured *ITGA6*-expression cells. For full gene list see Supplementary Figure 5Av. **(C)** Glucose- and fructose-adapted MCF-7 and MDA-MB-231 cells were transfected with the indicated siRNA 48 h prior to plating for mammosphere assay. Efficacy of the siRNA is demonstrated by RT-qPCR in Supplementary Figure 6. **(D)** Glucose- and fructose-adapted MDA-MB-231 cells were treated with c-KIT neutralizing antibody (0.2 μg/mL) 24 h prior to plating for mammosphere assay. Goat IgG was used as a negative control. **(E)** Glucose- and fructose-adapted MDA-MB-231 cells were treated with the indicated concentrations of imatinib mesylate 24 h prior to plating for mammosphere assay. (C, D, E) one way ANOVA and Fisher’s LSD test).

To examine the effects of glycolytic restriction at the molecular level in breast cancer cells, we initially performed RT-qPCR gene expression analysis with a panel of genes relevant to the SCLC cell phenotype (Figure 5A). The stem cell factor *c-KIT*, encodes a receptor tyrosine kinase; involved in self-renewal and therapeutic resistance in many cancers including breast [23]. Fructose-adapted MCF-7, ZR-75-1 and MDA-MB-231 cells exhibited an increased *c-KIT* expression as compared to the respective matched glucose cell pairs. *ITGA6,* which encodes the breast cancer stem cell marker CD49f [21, 23], was also modestly increased after fructose adaptation in MCF-7 and MDA-MB-231. Increased expression of Annexin A3 (*ANXA3*) correlates with SCLC cell phenotypes such as therapeutic resistance and increased migration [24], and its expression was increased by fructose-adaptation in MCF-7 and SKBR3 cells. In SKBR3 cells, fructose-adaption increased expression of dishevelled homologue 1 (*DVL1*), a WNT target gene known to be dysregulated in breast cancers [25]. Increased expression of *SLC7A5* (solute carrier family 7, member 5) in many cancers correlates with alternative cell survival strategies in the hostile tumor microenvironment [26]. Fructose-adapted SKBR3 cells exhibited an increase in *SLC7A5* expression as compared to the matched glucose cells, though *SLC7A5* was substantially decreased by fructose adaptation in MCF-7 cells. We did not see any significant effect of restricted glycolysis on the expression of *CTNNB1*, *BCL-2* or *BMI1* in any of the lines by this analysis.

These observed changes in gene expression substantiate our phenotypic evidence for altered SCLC cell profiles in response to decreased metabolism. To determine whether gene expression networks within SCLC cells were altered, we analyzed genome-wide mRNA expression in single-cells by the Drop-seq method [27]. This was performed in MDA-MB-231, as they demonstrated the largest phenotypic response to fructose adaptation, using cells cultured as mammospheres in order to enrich for SCLC cells. They also represent the triple negative subtype of breast cancer, for which research that may lead to novel therapeutic strategies is most urgent, due to the lack of biologically-targeted treatments for these tumors. Firstly, we identified genes differentially expressed in cells cultured in glucose or fructose (Supplementary Figure 4Ai). Pathway- and transcription factor binding site- analysis of these genes identified enrichment of glycolysis (p=3.6×10^-7^) and oxidative phosphorylation (p=2.9×10^-7^) pathways in glucose- versus fructose-cultured mammosphere cells respectively (Figure 5Bi & Supplementary Figure 5C). This confirmed that our experimental conditions retained the intended effects on cellular metabolism when the cells were cultured as mammospheres. An enrichment of genes with a HIF1 transcription factor binding site was also observed in the glucose-cultured cells (Supplementary Figure 5C, p=1.8×10^-2^), consistent with the known role of glycolytic metabolites in the hypoxia-independent regulation of HIF1 abundance [28]. Machine learning-based clustering analysis (using ‘metagenes’ of correlated gene sets identified by principle component analysis) on combined data from both sets of cells (Supplementary Figure 4Aii, 4Aiii), demonstrated the largest gene signature to be derived from cell cycle phase, as demonstrated by *MKI67* expression in cluster 3 (Supplementary Figure 4Aiv, p=9.3×10^-23^). Cells positive for *ITGA6* (CD49f) expression, one of the most frequently used markers of populations enriched in breast SCLC cells [21, 23] were distributed through both cycling and non-cycling clusters (Supplementary Figure 4Av). Expression of *c-KIT*, which can further define subsets of CD49f positive cells [23] and was increased by fructose adaptation in the prior RT-qPCR analysis of 2D-cultured cells, was below the resolution of this technique (∼10 molecules per cell [29]), consistent with relatively high Ct values in the c-KIT RT-qPCR in MDA-MB-231. To identify transcriptionally distinct subpopulations, irrespective of cell cycle phase at time of analysis, individual cells were assigned a cell cycle score which was used to regress the effects of cell cycle out of the data.

The subsequent modularity-based cluster analysis firstly clearly demonstrated that the culture conditions had a dominant role in determining the transcriptional profile of the cells (Figure 5Bii (insert) & Supplementary Figure 4Bi). The clustering analysis, with a medium resolution setting (res.0.75), partitioned cells into four clusters (Figure 5Bii and Supplementary Figure 4Bii) on which pathway and transcription factor binding site analysis was also performed (Supplementary Figure 5D). The largest cluster, 0^res0.75^, contained both glucose- and fructose-cultured cells, and expressed genes associated with chromatin reorganization pathway (p=3.3×10^-7^) and LEF1 transcription factor binding sites (p=1.0×10^-6^) (indicative of active Wnt signaling). Cluster 1^res0.75^ (mostly glucose-cultured cells) exhibited glycolysis pathway (p=5.7×10^-14^) and HIF1 binding sites (p=3.1×10^-3^), whereas cluster 2^res0.75^ (mostly fructose-cultured cells) had no significant enrichment of pathway-related genes, but had a reduced LEF1 binding site signature (p=2.1×10^-4^). The smallest cluster, 3^res0.75^ (containing cells from both culture conditions, but enriched in fructose-cultured) upregulated signatures of translation elongation (p=3.3×10^-11^) and AP1 binding sites (p=2.4×10^-2^). *ITGA6* positive cells were concentrated in clusters 0^res0.75^ and 3^res0.75^ (Figure 5Biii) suggesting these represent two distinct pools of cells enriched in SCLC cells. Reinforcing this; the most significant (p=3.5×10^-25^) individual marker of cluster 0^res0.75^ is *ITGB1* (CD29, a well-documented breast SCLC cell marker [23]), and of cluster 3^res0.75^ is *HMGA1* (p=1.8×10^-15^), a driver of the stem-like state in triple negative breast cancer cells [30] (Figure 5Biii). Also, notable in cluster 0^res0.75^ was a highly significant enrichment (p=6.8×10^-133^) of genes associated with the NGF-TrkA growth factor tyrosine kinase signaling pathway [31]. The glucose-cultured cells represent the mammosphere culture conditions closest to much of the literature, therefore we also examined these cells in isolation, based on the above clusters (Supplementary Figure 5Bi & 5E). Notably, the cluster with the lowest proportion of *ITGA6* positive cells, cluster1^res0.75^, still demonstrated a signature of high glycolysis compared to the other clusters, despite all the cells being in the same media (p=6.6×10^-8^) (Supplementary Figure 4Biii). This is consistent with this cluster representing the bulk tumor cells, which have been demonstrated to be more reliant on glycolysis than the more oxidative breast SCLC-enriched populations [3].

To determine whether the transcriptional profile of the cells within the two *ITGA6*-enriched clusters is affected by restriction of their glycolytic metabolism, the clustering analysis was repeated at a higher resolution setting (res.1) (Figure 5Biv and Supplementary Figure 4Biv) to identify further transcriptionally distinct cell communities. Whilst the smaller, fructose-cultured-cell dominated cluster 3^res0.75^ did not further resolve at this or higher resolutions (res.>1), cluster 0^res0.75^ was essentially resolved into two clusters 0A^res1^ (predominantly glucose-cultured cells) and 0B^res1^ (almost exclusively fructose-cultured cells). Cells in both clusters exhibited comparable expression of *ITGA6* (Supplementary Figure 4Bv). Compared to 0A^res1^, 0B^res1^, showed an increased signature for respiratory electron transport (p=4.2×10^-11^) (e.g. *ATP6V0E1*, Supplementary Figure 4Bv), whereas signatures for SOX9 (p=5.7×10^-3^) and LEF1 (p=9.3×10^-3^) transcription factor binding sites and NGF-TrkA signaling (p=1.1×10^-77^) were higher in cluster 0A^res1^ (Supplementary Figure 5F). As a complementary approach to determine the effect of glycolytic restriction on the SCLC cells, the entire cell dataset was down-sampled and filtered based on positive *ITGA6* expression and reanalyzed; (Figure 5Bv, Supplementary Figure 4C & 5G). Compared to their glucose-cultured counterparts, fructose-cultured *ITGA6* positive cells exhibited a significant (p=1.2×10^-4^) upregulation of genes in the oxidative phosphorylation pathway including *NDUFB1*, *COX6B1*, *UQCRG* and *COX8A*, whereas the glycolysis pathway (e.g. *PGK1*) was significantly downregulated (p=1.5×10^-9^). The LEF1 transcription factor binding site signature and NGF-TrkA signaling signature were both significantly higher in the glucose-cultured cells (p=3.2×10^-3^ and 5.9×10^-13^ respectively). Other specific genes which showed decreases in expression in the glycolysis-restricted cells include *EMP1* and *EHF*, both of which are markers of mammary stem cell differentiation programs [32, 33]. Thus both of these analytical approaches of identified potential SCLC cell-enriched populations and demonstrated that restricting glycolysis did result in altered metabolism, expression of stem cell pathways markers, and substantial changes in intracellular growth factor signaling pathways within these cells.

Having demonstrated that the SCLC cells in which glycolysis has been restricted have reprogrammed their expression of metabolic genes, we next examined whether this resulted in a changed dependency on specific metabolic pathways. ACSS2 metabolism has been shown to be a critical gene for cancer cell survival under conditions of metabolic stress, as it facilitates the use of acetate as a nutritional source [34]. Transfection of cells with siRNA to *ACSS2* caused a significant decrease in the mammosphere forming ability of fructose-adapted MCF-7 and MDA-MB-231 cells respectively (Figure 5C), whereas it had the opposite effect in glucose-cultured cells. Thus, the SCLC cells in the two culture conditions are indeed dependent upon demonstrably distinct metabolic pathways.

The effect of glycolytic restriction on gene expression in SCLC cell-enriched populations expressing *ITGA6* extended beyond metabolic genes to pathways that impact on broader cell phenotypes. c-KIT is a known marker of distinct populations of *ITGA6* (CD49f) positive breast stem and progenitor cells [23]. As the RT-qPCR analysis detected increased *c-KIT* expression in the fructose-cultured cells, this could represent a metabolism-associated change in the phenotype of the SCLC cells. Given that c-KIT is one of a small number of SCLC cell markers that can be required for survival and proliferation of the cells [35], this hypothesis was readily testable. We found *c-KIT* siRNA had a negative impact on mammosphere formation in both glucose- and fructose-adapted MCF-7 cultures, whereas, strikingly, in MDA-MB-231 it only had a negative impact on mammosphere formation in the fructose-adapted cultures (Figure 5C). Moreover, neutralization of c-KIT function by c-KIT antibody, or functional inhibition of c-KIT tyrosine kinase by imatinib, in glucose- and fructose-adapted MDA-MB-231 cells corroborate the results from the siRNA experiment (Figure 5D and 5E). Mechanistically, the single-cell mRNA-seq data provides a number of potential insights for the differential c-KIT dependence; TrkA-related signaling pathways, which were significantly higher in the glucose-cultured cells, can render the c-KIT signaling functionally redundant and hence cause imatinib resistance [36], or an elevated proliferative drive from WNT signaling in the glycolytic cells could have a comparable effect. Furthermore, *EMP1*, expression of which was higher in the glucose-cultured *ITGA6* positive cells, is a biomarker of resistance to another TKI, gefitinib, in lung cancer [37].

## DISCUSSION

We have demonstrated that restriction of glycolysis in cell lines derived from breast cancers of different subtypes, consistently results in an increase in the SCLC cell population. From this, combined with our single-cell RNA-seq analysis, we can surmise that, whilst these tumor-derived cells presumably contain genetic changes that predispose them to high rates of glycolysis when glucose is available [38], the SCLC cells in the population retain the potential to adapt to a less glycolytic and more oxidative state in response to reduced availability of glucose. This potential is consistent with Cuyas *et al* [39], who showed that enrichment of HMLER breast cancer cells for stem-like properties by inducing epithelial-mesenchymal transition, resulted in their ability to metabolize a wider range of substrates, including fructose. The presence of chemoresistant oxidative SCLC cells in glucose-deprived conditions has been previously proposed [4], and indeed supported from experiments with glucose-starved ovarian carcinoma cells [40], however it has been inferred from these and similar studies [3], that such cells exist in a slow-cycling or quiescent state. What is clear from the experimental data we present here is that such non-glycolytic cells can in fact be highly proliferative.

Recent reports examining the metabolic state of SCLC cells have concluded that they can be either more glycolytic or more oxidative than the bulk tumor cell population [3, 4, 6]. Such different conclusions can potentially be explained by the methods used to enrich for the stem cell populations; for example Feng *et al* [8] enriched for breast SCLC cells by isolating the mesenchymal/basal CD49f^high^ Epcam^low^ population, and found these to be more glycolytic than the CD49f^low^ Epcam^high^ luminal cells, which contained few stem cells. This, therefore, may indicate an association between glycolysis and cells exhibiting a mesenchymal phenotype, rather than with a stem-cell like phenotype *per se*. Culture as non-adherent spheroids is one of the established methods of SCLC cell enrichment [41]. Yuan *et al* [42] and Ciavardelli *et al* [43], took stem-cell enriched spheroid cultures from glioblastoma and breast, and allowed them to differentiate into monolayer by the addition of serum. As the cells differentiated, glycolysis decreased and oxygen consumption increased, implying that the SCLC cells preferentially rely on glycolytic metabolism. In contrast, in other studies of breast cancer [44], pancreatic cancer [45] and glioma [46], cell lines maintained in 2D cultures were plated as single cells into low attachment conditions to form mammospheres. The stem cell-enriched floating spheres were then compared with attached bulk cell populations and were found to have a more oxidative phenotype. One possible explanation for these differential findings is that the short-term response to detachment from the extracellular matrix (ECM) is downregulation of glucose uptake and consequent reactive oxygen species (ROS)-dependent death of the bulk cell population [47]. However over long-term culture, spheroids develop an hypoxic core and upregulate HIF1α [48], which promotes glycolysis and protects from ROS [49]. Indeed, HIF1α was shown to be elevated in the mammospheres in the Ciavardelli study [43]. This dichotomy is replicated when one compares the effects of distinct metabolic interventions on the enrichments of SCLC cells in standard 2D cultures, on one hand adaptation to conditions which stably promote oxidative over glycolytic metabolism enrich for SCLC cells (this study), whereas on the other hand hypoxia, which promotes glycolysis, also enriches for SCLC cells [41, 49]. Both of these adaptations would render the cells less dependent on ECM-attachment associated glycolysis for their maintenance of redox homeostasis, and thus increase survival under non-adherent conditions in stem cells assays.

Importantly, as discussed by Semenza [49], in addition to merely enhancing SCLC cell maintenance by protecting from metabolic stress, HIFs also specify the SCLC cell-state through promoting the expression of genes such as *NANOG*. Similarly, here we have shown the restriction of glycolysis and promotion of a more oxidative state increases the expression of both stem cell markers such as CD44 and ALDH, as well as genes know to be involved in specifying the stem cell-like state such as *c-KIT* [50]. It also markedly alters the gene expression profiles of the *ITGA6* (CD49f) positive SCLC cell-enriched populations in the mammospheres. In the normal (murine) breast c-KIT is a particularly informative marker, as is only expressed on a subset of early luminal progenitor cells, whereas mammary stem cells and more mature progenitors/ differentiated cells are c-KIT^low/-ve^ [23, 35]. c-KIT positive cells can be the cell of origin in some ER^-ve^ /HER2^-ve^ breast cancers [35], and c-KIT expression is particularly associated with the triple negative subtype. Our data from the ER^-ve^ MDA-MB-231 supports a model in which the glycolytic SCLC cells can take on characteristics of either c-KIT^+ve^ early progenitors or c-KIT^low^ late progenitors, whereas under glycolysis-restricted conditions they are more constrained to the c-KIT^+ve^ early progenitor-like state and are therefore more c-KIT dependent. ER^+ve^ cells appear not to exist in a c-KIT^low^ progenitor state, [23], and our finding that ER^+ve^ MCF-7 SCLC cells are c-KIT dependent irrespective of their glycolytic state is consistent with this. It is possible that this ER^-ve^ SCLC plasticity is a feature of subsets of cancer cells, accounting for the observations that *c-KIT* knockdown effectively eliminates the 3D culture potential of primary mammary cells [35], whereas therapies targeting c-KIT in breast cancer patients have had limited success [51].

The metabolic profile of the cells within a tumor is likely to vary temporally and spatially during tumor development, in response to changing cell-intrinsic factors such as oncogenic mutation, and cell-extrinsic influences from the micro-environment [38]. Recognition of this metabolic plasticity in tumor cells is leading to the development of therapeutic strategies to obviate therapeutic resistance to targeting a single pathway [6, 8]. Given the role of SCLC cells in resistance to therapy, it is critical to understand the role of metabolic plasticity in defining the SCLC phenotype. Recent work has demonstrated that SCLC cells can be either more, or less, glycolytic than their bulk tumor counterparts. Studies of cellular responses to hypoxia have demonstrated increased glycolysis can be causative of, rather than merely correlative with, the stem cell phenotype [49]. Here we have shown that adaptation of cells to a less glycolytic, more oxidative metabolism can also be a causative factor in the development and phenotype of SCLC cells, with important implications for the development and treatment of malignancies of epithelial origin.

## MATERIALS AND METHODS

### Cell culture

Cell lines stocks (MCF-7 [ATCC^®^ HTB-22^™^], ZR75-1 [ATCC^®^ CRL-1500^™^], SKBR3 [ATCC^®^ HTB-30^™^] and MDA-MB-231 [ATCC^®^ HTB-26^™^] were validated by STR profiling (DDC Medical) and mycoplasma testing. Cell lines were adapted for > 30 days in DMEM containing either 25 mM glucose or 10 mM fructose (base medium #D5030 Sigma, U.K. with sodium pyruvate 1 mM, L-glutamine 2 mM, sodium bicarbonate 3.7 g/L, Penicillin (100 U/mL) / Streptomycin (100 μg/mL) and 10% fetal calf serum). Transfection with 5 nM of Silencer^®^ select siRNA; c-KIT (ID: s7869) or ACSS2 (ID: s31746) or universal negative control (Ambion, Thermo Fisher Scientific, UK) used INTERFERin^®^ (Polyplus-transfection^®^, France), 48 hours prior to assay. Paclitaxel and Imatinib mesylate were from Sigma Aldrich, UK. Human CD117/c-KIT antibody (AF332) and IgG control from R&D Systems, UK. All photo-microscopy of cells was performed using an Olympus IX81 microscope-based system equipped with Xcellence Pro software.

### Extra cellular acidification rate (ECAR), oxygen consumption rate (OCR) and ATP

ECAR and OCR were measured using Seahorse Bioscience XF96 Extracellular Flux Analyzer (Seahorse Bioscience, MA). Manufacturer’s protocols were used except glucose (5 mM) was substituted with fructose (5 mM) in the fructose-adapted cells. Data were normalized to total protein per well. ATP was measured by ATPlite Luminescence Assay System (Perkin Elmer, Boston, MA).

### Cell function assays

Organization of F-actin: cell monolayers were fixed in ice-cold 4% paraformaldehyde in PBS for 10 min. After blocking in 0.1% Triton-X-100, 0.2% BSA in PBS for 40 min., cells were incubated in 5 mg/l phalloidin-FITC (Sigma-P5282) in PBS for 40 min. Nuclei were stained with DAPI (Sigma-D9564). 3D matrigel cell growth: 96-well culture dishes were coated with matrigel (VWR) at 37°C for 30 minutes. Cells seeded in 4% matrigel-containing media, containing the appropriate sugar. After 7-8 days, cell proliferation was measured by alamarBlue (Life Technology, CA, USA). Cell invasion: 8 μm Transwell inserts were coated with matrigel (1:30 in serum free media). Cells were then seeded (in serum free media) onto the inserts, which were then incubated in the culture well in serum containing media for 24-48 hours. Non-invaded cells were removed by gently rubbing the inner layer of the membrane using a cotton swab, and invaded cells on the outer membrane were fixed with ice cold 4% paraformaldehyde, stained with Hoechst stain, and counted by fluorescence microscopy. Invasion efficiency was calculated as (number of cells invaded through the inserts / total number of seeded cells) X 100 Colony assay: cells were seeded at 10000 cells/ well in six well plate format. After overnight incubation, media was replaced with media containing either DMSO carrier control or paclitaxel (5 nM) and cells allowed to grow for 8-10 days. Colonies were then stained with 0.1% crystal violet, which was quantified by dissolving in 20% acetic acid and measuring absorbance at 595 nm.

### Mammosphere culture

2000 cells were seeded in 100 μl DMEM:F12-based mammosphere media conditions (supplemented with 20 ng/mL recombinant human EGF, 20 ng/mL recombinant human basic FGF, B27 supplement, 0.4% FCS, penicillin-streptomycin, L-glutamine (all from Life Technology, USA) and 5 μg/mL bovine insulin (Sigma)) on polyhema coated low attachment 96-well plates with the sugar in the mammosphere cultures being the standard 17.5 mM glucose in DMEM:F12 for all assays. After 8-10 days, representative images were captured by microscopy (bar 100 μm) and mammospheres were measured by alamarBlue. For harvesting glucose and fructose-cultured mammospheres for Drop-Seq analysis, the mammosphere media was substituted with conditioned media from the respective 2D cultures.

### ALDEFLUOR assay

The ALDEFLUOR assay kit from Stem Cell Technologies, UK was used. Cells were incubated with ALDEFLUOR substrate (BAAA, BODIPYaminoacetaldehyde. Stem Cell Technologies, UK) to define the ALDEFLUOR-positive cells, and a specific inhibitor of ALDH1, diethylaminobenzaldehyde (DEAB), was used to establish the baseline fluorescence. Flow cytometry plots indicate side scatter (SSC) versus fluorescence intensity.

### CD24/CD44 cell surface markers staining

Briefly, respective matched pairs of either glucose- or fructose-adapted breast cancer cells were incubated with cell surface marker fluorescent antibodies (CD24-FITC 32D12 and CD44-APC DB105, Miltenyi Biotec, UK) and the percentage of CD44^high^/CD24^low/-ve^ cell population was calculated.

### Immunoblotting

For western blotting, equal amount of proteins were initially boiled in Tris-lysis buffer and electrophoresed on a 10% SDS-polyacrylamide gel. Proteins were transferred onto nitrocellulose membrane (VWR) and followed by blocking in 5% milk (in 0.1% PBS-Tween-20). Blots were probed for lactate dehydrogenase A (LDHA) (C4B5. Cell Signaling Technology (Beverly, MA) was visualized with SuperSignal (ThermoFisher Scientific), using the appropriate secondary antibody. Rabbit polyclonal anti-β-actin antibody (Sigma, St Louis, MO) was used as a loading control.

### Quantitative PCR

Total RNA was extracted from cell lines using Reliaprep™ RNA cell miniprep system (Promega, USA). RT-qPCR was performed using Taqman^®^ universal PCR mastermix (ThermoFisher Scientific) with Roche Universal probe library assays (Roche, Germany), using the following primer pairs and probes numbers: *ANXA3*: TCCGGAAAGCTCTGTTGACT/ATCTTGTTTGGCCAGATGCT/#29; *CTNNB1*: GCTTTCAGTTGAGCTGACCA/CAAGTCCAAGATCAGCAGTCTC/#21; *DVL1*: AAGAACGTGCTCAGCAACC/AGCTTGGCATTGTCATCAAA/#63; *BCL-2*: GTACCTGAACCGGCATCTG/GGGGCCATATAGTTCCACAA/#75; *SLC7A5*: GTGGAAAAACAAGCCCAAGT/GCATGAGCTTCTGACACAGG/#25; *c-KIT*: CTTTCCTCGCCTCCAAGAAT/GTGATCCGACCATGAGTAAGG/#71; *BMI1*: CCATTGAATTCTTTGACCAGAA/CTGCTGGGCATCGTAAGTATC/#63; *ACSS2*: CCCCAATTAAGAGGTCATGC/CACTCGGGCTCACACTCAT/#34); *ITGA6*: ATTCTCATGCGAGCCTTCAT/GGAAACACAGTCACTCGAACC/#74. Expression was normalized to β-actin (ThermoFisher Scientific Taqman^®^ assay 4326315E) using the ΔΔCt method. n=3 biological repeats. mRNA expression in fructose-cultured cells is shown relative to the expression in glucose-cultured cells. Statistical analysis (paired *t*-test) was performed on ΔCt values.

### Whole transcriptome single-cell mRNA sequencing (Drop-Seq)

Drop-seq utilizes a custom microfluidic platform designed to encapsulate cells in nanolitre droplets along with DNA-barcoded beads (https://dropletkitchen.github.io/), followed by highly parallel analysis of individual cells by RNA-seq. Experiments were performed according to Macosko *et al.* [27] with the following adjustments. Single-cells (100 cells/ul) and barcoded mRNA-binding micro-particles (100 beads/ml) were suspended in droplets containing cell lysis buffer (∼1 nl; 50 cell /ml final concentration). Droplets were then broken and collected by centrifugation and subjected to cDNA synthesis (Maxima H- RTase), introducing the molecule and cell barcode to every transcript from a single cell (termed a ‘STAMP’). 800 STAMPs from each condition were then selected for PCR amplification (13 cycles), library preparation (Nextera XT, Illumina; using 500 pg cDNA) and Illumina sequencing by synthesis using a custom read 1 primer (NextSeq-500 platform; version 2 chemistry - high output setting; 20 bp read 1, 50 bp read 2 and an 8 bp index 1). Species-mixing experiments are routinely performed in our laboratory and have determined that our implementation of the Drop-Seq protocol robustly achieves single-cell encapsulation and captures transcriptomes from single-cells with high specificity (98.5% of cell encapsulation events are single-species; data not shown). Raw sequencing reads were converted to a sorted unmapped BAM file (FastqToSam, Picard bundled in Dropseq-tools v1.2; http://mccarrolllab.com/dropseq) and filtered to remove all read-pairs with a barcode base quality of <10. The second read was trimmed at the 5’ end to remove any TSO-adapter sequence and at the 3’ end to remove polyA tails. Reads were aligned against human reference genome (hg19) using STAR aligner (v2.5.0a), then sorted/converted/merged to a BAM with a tag ‘‘GE’’ onto reads for data extraction. The DigitalExpression program (Dropseq-tools v1.2) performed digital counting (DGE) of the mRNA transcripts (unique molecular identifiers to avoid double counting reads/PCR duplicates) and created a DGE matrix (one measurement per gene per cell). Analysis of the DGE matrix was performed in Seurat (Seurat: R toolkit for single cell genomics. R package version 2.0; MS windows). To exclude low quality cells and likely cell doublets, cell barcodes with fewer than 2000 genes and greater than 15,000 UMIs were removed. All genes that were not detected in at least 3 cells were discarded, and all mitochondrial DNA-encoded genes were excluded, leaving 13,682 genes across 303 cells (128 glucose, 178 fructose). The digital gene expression matrix was library-size normalised, scaled by the total number of transcripts, multiplied by 10,000 and natural-log transformed before further downstream analysis with Seurat. For sub-setting on *ITGA6* expressing cells (34 glucose, 61 fructose), fructose-cultured cells were randomly down-sampled in Seurat. All Seurat script used are provided in Supplementary Figure 5. Gene list enrichment analysis was performed using ToppGene Suite, [52] and Bonferroni-corrected p values reported. The sequencing data have been deposited to the gene expression omnibus database (https://www.ncbi.nlm.nih.gov/geo/) with identifier [GSE106202].

### Orthotopic tumor xenograft in nude mice

Animal work was done in accordance with a protocol approved by the University of Southampton Animal Welfare and Ethical Review Body and under Home Office license PB24EEE31. Initially, serial low dilutions of MDA-MB-231-glucose cells were injected in the mammary fat pad of NOD/SCID mice to identify the lowest cell numbers that cause visible tumor growth within six weeks. Based on this, 1.5 × 10^4^ and 3 × 10^4^ cells of either glucose- or fructose-adapted MDA-MB-231 were injected on both flanks in six mice, respectively. Tumor volume was calculated using the formula (length X width^2^)/2. Stem cell frequency was estimated using ELDA [53].

### Data analysis and statistical methods

Data for the cell assays and RT-qPCR were analyzed in Microsoft Excel and GraphPad Prism. Unless stated otherwise data are mean ± SEM of biological triplicates (n=3, each with ≥ 2 technical replicates), and expressed as relative to the means from glucose-cultured cells. Unless stated otherwise, statistical tests are paired, two sided, *t*-tests. For all statistical analysis: ^***^ P<0.001, ^**^ P<0.01, ^*^ P<0.05.

## SUPPLEMENTARY MATERIALS FIGURES



















## Abbreviations

SCLC: stem cell-like cancer
ECAR: Extracellular acidification rate
OCR: oxygen consumption rate
BAAA: BODIPYaminoacetaldehyde
DEAB: diethylaminobenzaldehyde
SSC: side scatter
ALDH: aldehyde dehydrogenase
ECM: extracellular matrix
ROS: reactive oxygen species
