# Supplementary material for: The effects of restricted glycolysis on stem-cell like characteristics of breast cancer cells
Source: Oncotarget. 2018 May 1;9(33):23274–88. doi: 10.18632/oncotarget.25299 (PMC5955399; doi:10.18632/oncotarget.25299)
Supplement: Supplementary file 1 [file oncotarget-09-23274-s001.pdf]

The effects of restricted glycolysis on stem-cell like characteristics of breast cancer cells

SUPPLEMENTARY MATERIALS

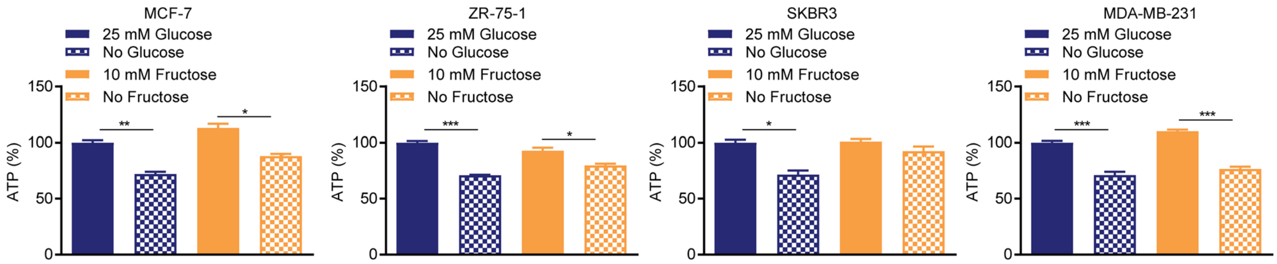

Supplementary Figure 1: Dependence of fructose-adapted cells on fructose for ATP maintenance. In one of the ATP assays described in Figure 1, the effect of transferring fructose-adapted cells to media containing 0 mM fructose for 24 h was also tested. n=3 technical replicates.

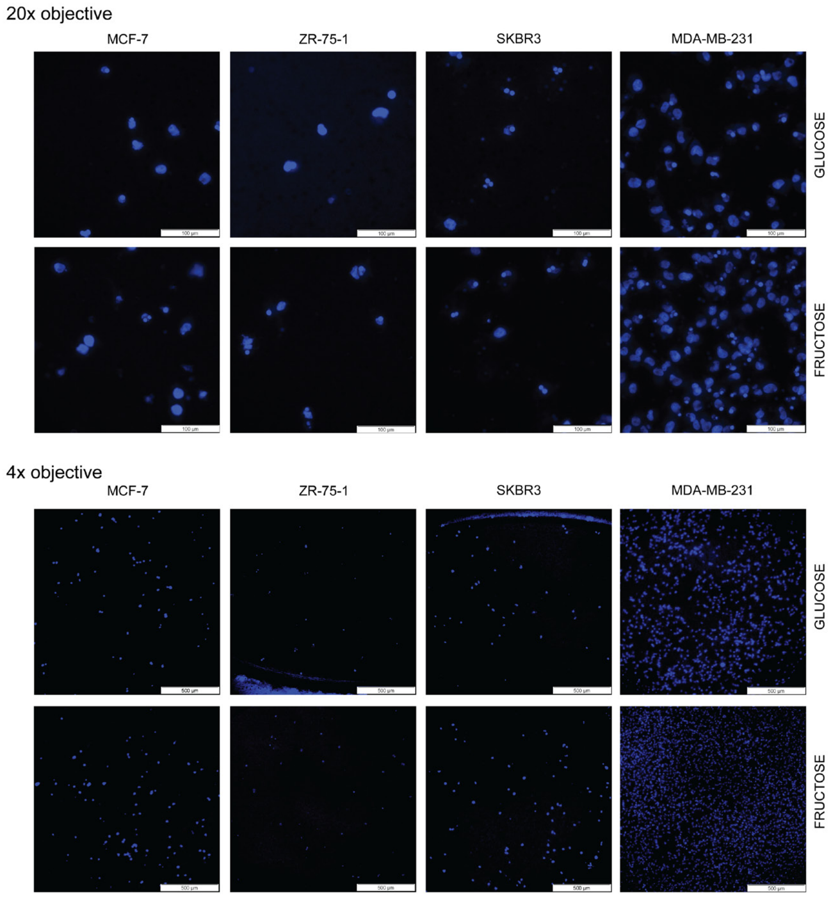

Supplementary Figure 2: Restricted glycolysis alters invasiveness in breast cancer cells. Invasion assays were performed as in Figure 2E. Photo-microscopy was performed on representative fields using either a 20 X objective (bar = 100 µM) or 4 X objective (bar = 500 µM).

	Weeks Tumours		
# of cells	4	5	6
1e6	2 of 2	2 of 2	2 of 2
3e5	2 of 2	2 of 2	2 of 2
1e5	1 of 2	1 of 2	1 of 2
3e4	0 of 2	2 of 2	2 of 2
1e4	0 of 2	0 of 2	0 of 2
3e3	0 of 2	0 of 2	0 of 2

Supplementary Figure 3: MDA-MB-231 orthotopic xenograft model: limiting cell dilution analysis. Serial dilution ($1x10^6$ to $3x10^3$) of MDA-MB-231 (glucose-adapted) cells were injected on both flanks into mammary fat pad areas (n=2) of six week-old female SCID/NOD mice (Charles River Laboratories, UK) and tumor initiating capacity was observed for a period of six weeks. All animal work was done in accordance with a protocol approved by the Institutional Animal Care and Ethics Committee of the Faculty of Medicine, University of Southampton. PIL: I8EA75E4C.

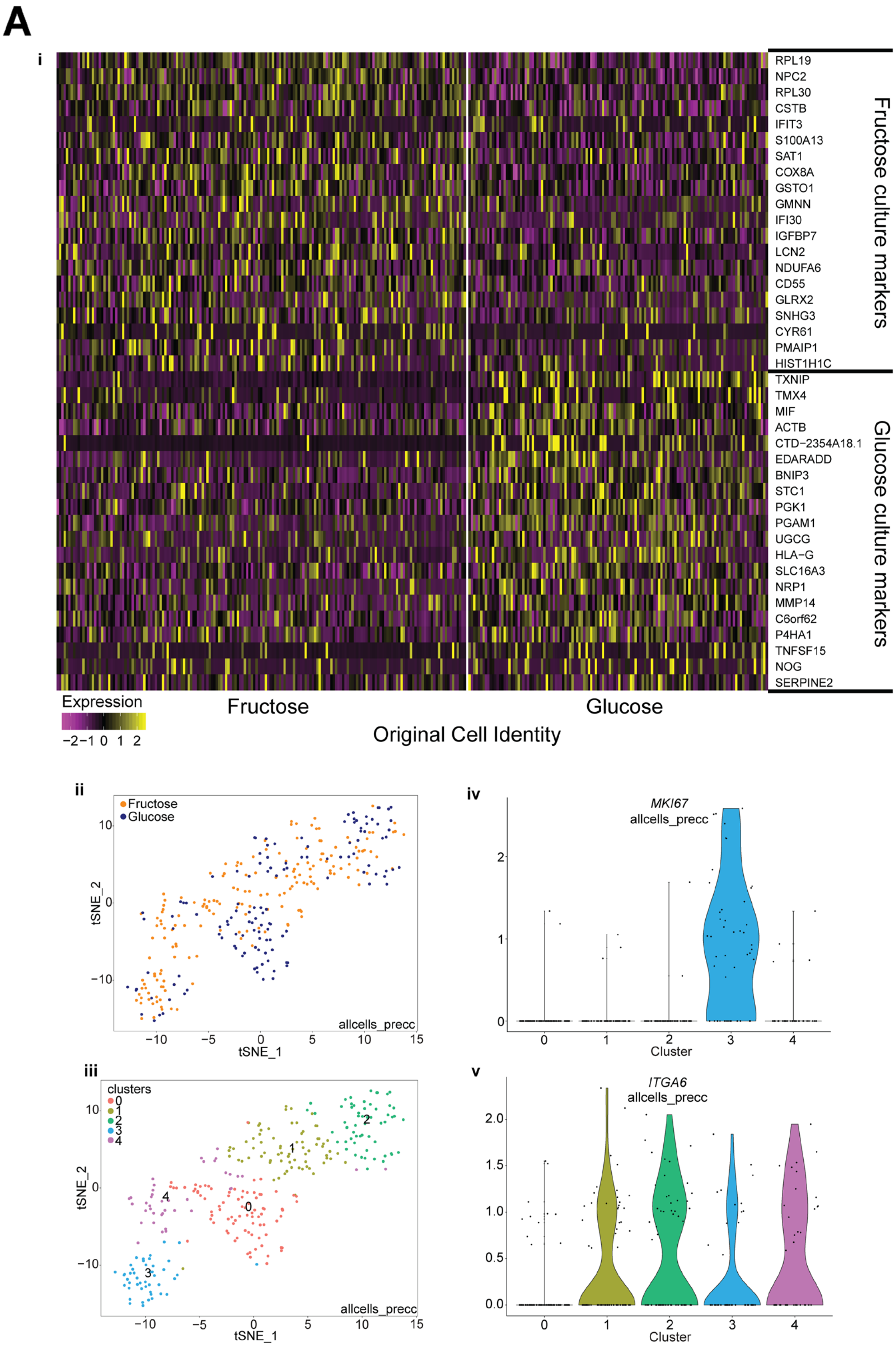

Supplementary Figure 4: Single cell mRNA-seq (Drop-Seq) analysis of mammosphere-cultured MDA-MB-231 cells. (A) Drop-seq analysis of MDA-MB-231 mammospheres prior to regressing out of cell cycle scores. Generated by R script Seurat_ GLUFRU_precc.R (Supplementary File 5_Scripts). (i) heatmap of variable gene expression between glucose and fructose-cultured cells. For full gene list and functional analysis see Supplementary File 5Ai and 5C. (ii) t-SNE plot showing original cell identity. (iii) t-SNE plot showing clustering. (iv) violin plot of *MKI67* (Ki67) expression demonstrating the enrichment of cycling cells in cluster 3. (v) violin plot of *ITGA6* (CD49f) expression demonstrating that this SCLC cell marker is not restricted to cycling cells.

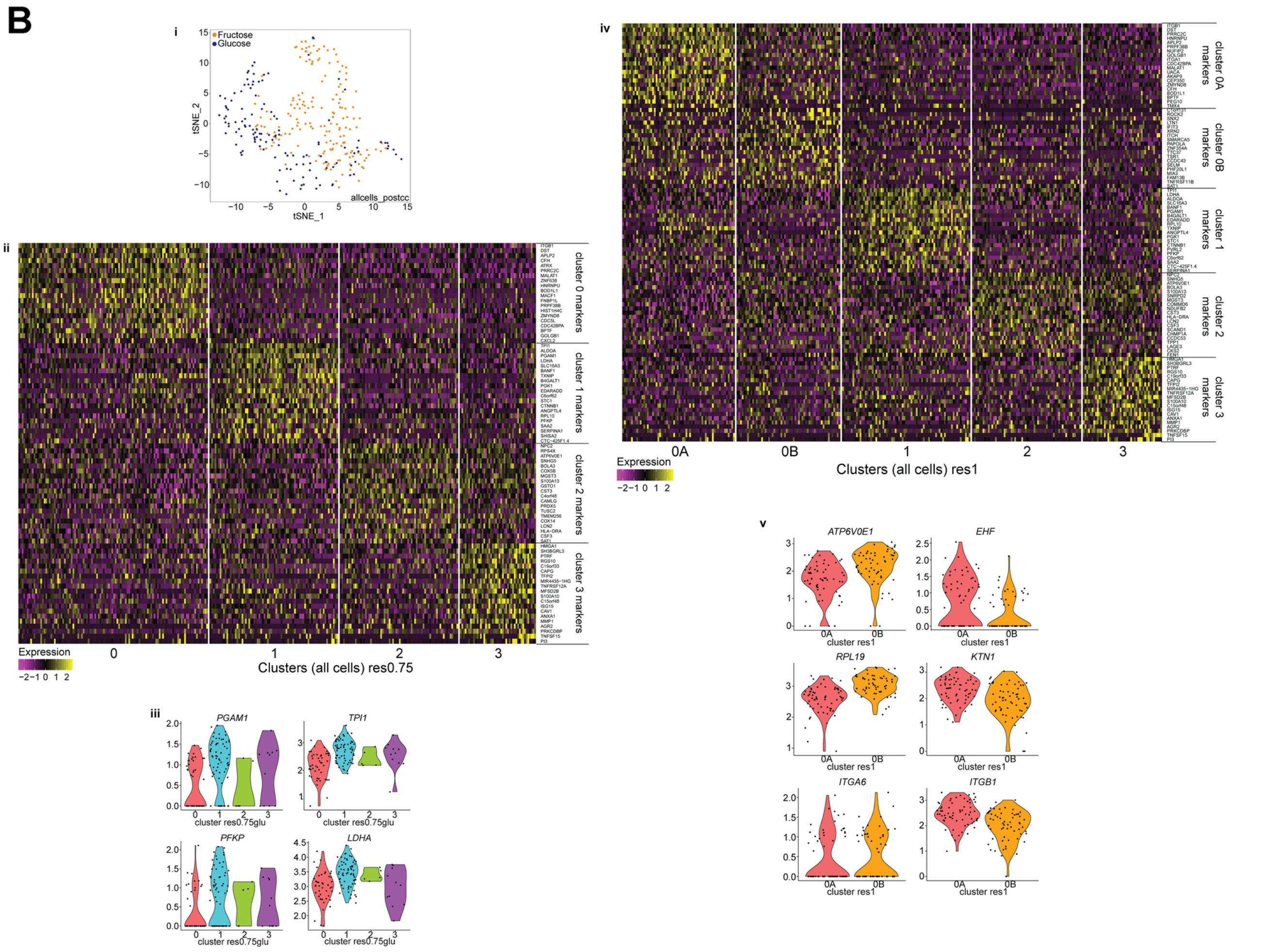

Supplementary Figure 4 (*Continued*): Single cell mRNA-seq (Drop-Seq) analysis of mammosphere-cultured MDA-MB-231 cells. (B) Drop-seq analysis of MDA-MB-231 after regression out cell cycle scores. Generated by R script Seurat_GLUFU_postcc.R (Supplementary File 5_Scripts). (i) t-SNE plot showing original cell identity. (ii) Heatmap of variable gene expression between clusters, with clustering performed at medium resolution (res.0.75). For full gene list see Supplementary File 5Aiii. (iii) Violin plot comparing the expression of representative glycolytic genes in glucose cells within the clusters (res0.75) (iv) Heatmap of variable gene expression between clusters, with clustering performed at higher resolution (res.1). For full gene list see Supplementary File 5Aiv. (v) Violin plot comparing the expression of representative genes in cells from clusters 0A and 0B at res.1.

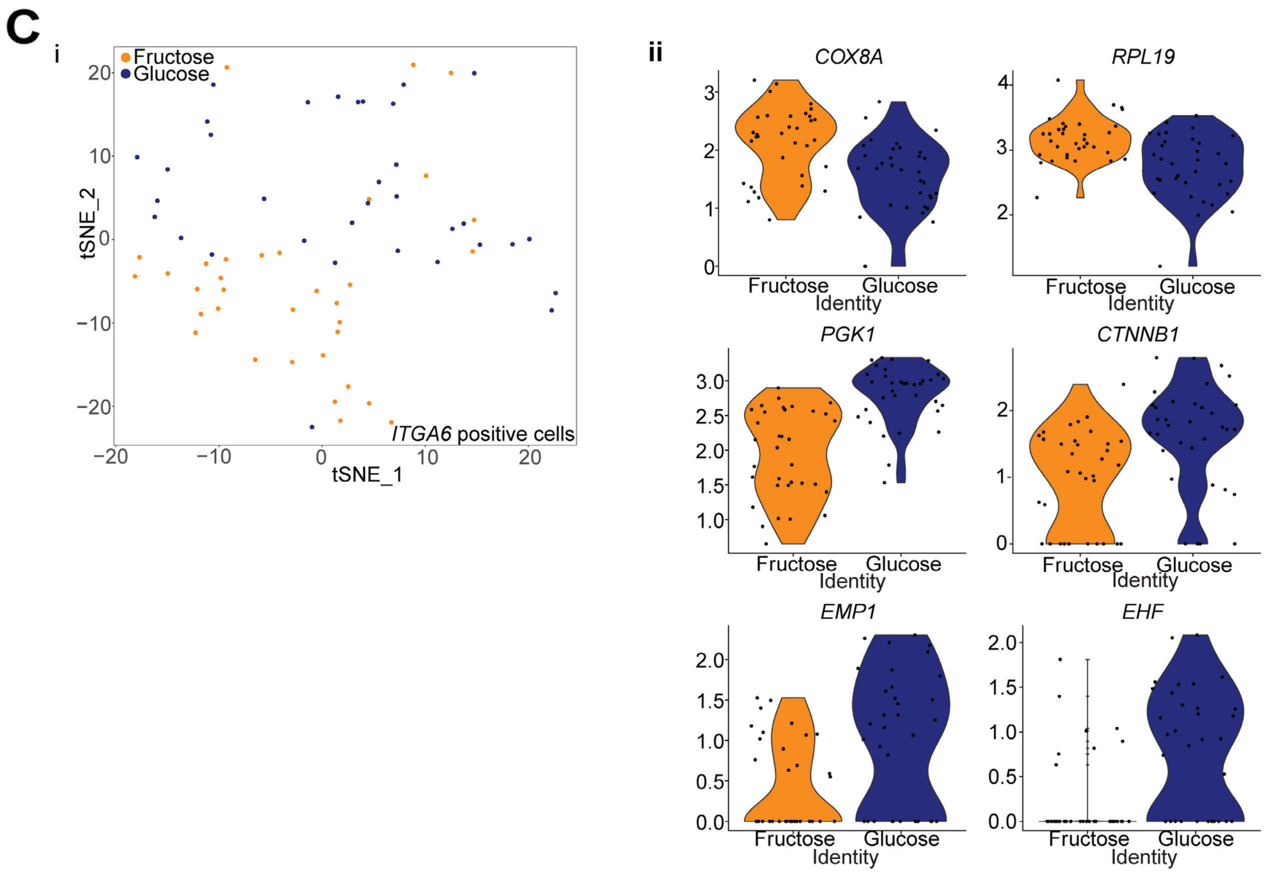

Supplementary Figure 4 (*Continued*): Single cell mRNA-seq (Drop-Seq) analysis of mammosphere-cultured MDA-MB-231 cells. (C) Drop-seq analysis of *ITGA6* positive MDA-MB-231 cells. Generated by R script Seurat_GLUFRU_postcc_ITGA6.R (Supplementary File 5_Scripts) (i) t-SNE plot showing original cell identity (ii) Violin plot of representative genes differentially expressed between *ITGA6* positive cells in fructose versus glucose mammosphere media.

Supplementary Figure 5: Data for single cell mRNA seq. analysis.

Text file containing three R scripts used for the analysis of Drop-seq data from MDA-MB-231 cells cultured as mammospheres in media containing either 25 mM glucose or 10 mM fructose.

Digital gene expression matrix file used as source data for the scripts is deposited at https://www.ncbi.nlm.nih.gov/geo/ and assigned the identifier [GSE106202]).

See Supplementary File 1

Supplementary File 5A: Markers generated by Function [FindAllMarkers(test.use="bimod")]

Gene lists:
i) allcells_origident
(ii) allcells_precc_clust
(iii) allcells_postcc_clustr.75
(iv) allcells_postcc_clustr1
(v) ITGA6cells_postcc_origident

See Supplementary File 2

Supplementary File 5B: Markers generated by Function [FindMarkers(test.use="roc")]

(i) GLUcells_postcc_r.75_1vALL
(ii) allcells_postcc_r1_0Bv0A

Gene list enrichment analysis and candidate gene prioritization based on functional annotations and protein interactions network [https://toppgene.cchmc.org/enrichment.jsp]

See Supplementary File 3

Supplementary File 5C: Analysis of Ai

Glucose up
Fructose up

See Supplementary File 4

Supplementary File 5D: Analysis of Aiii

cluster 0 up
cluster 1 up
cluster 2 up
cluster 3 up

See Supplementary File 5

Supplementary File 5E: Analysis of Bi

up in 1vALL

See Supplementary File 6

Supplementary File 5F: Analysis of Bii

up in 0Bv0A
down in 0Bv0A

See Supplementary File 7

Supplementary File 5G: Analysis of Av

fructose up
glucose up

See Supplementary File 8

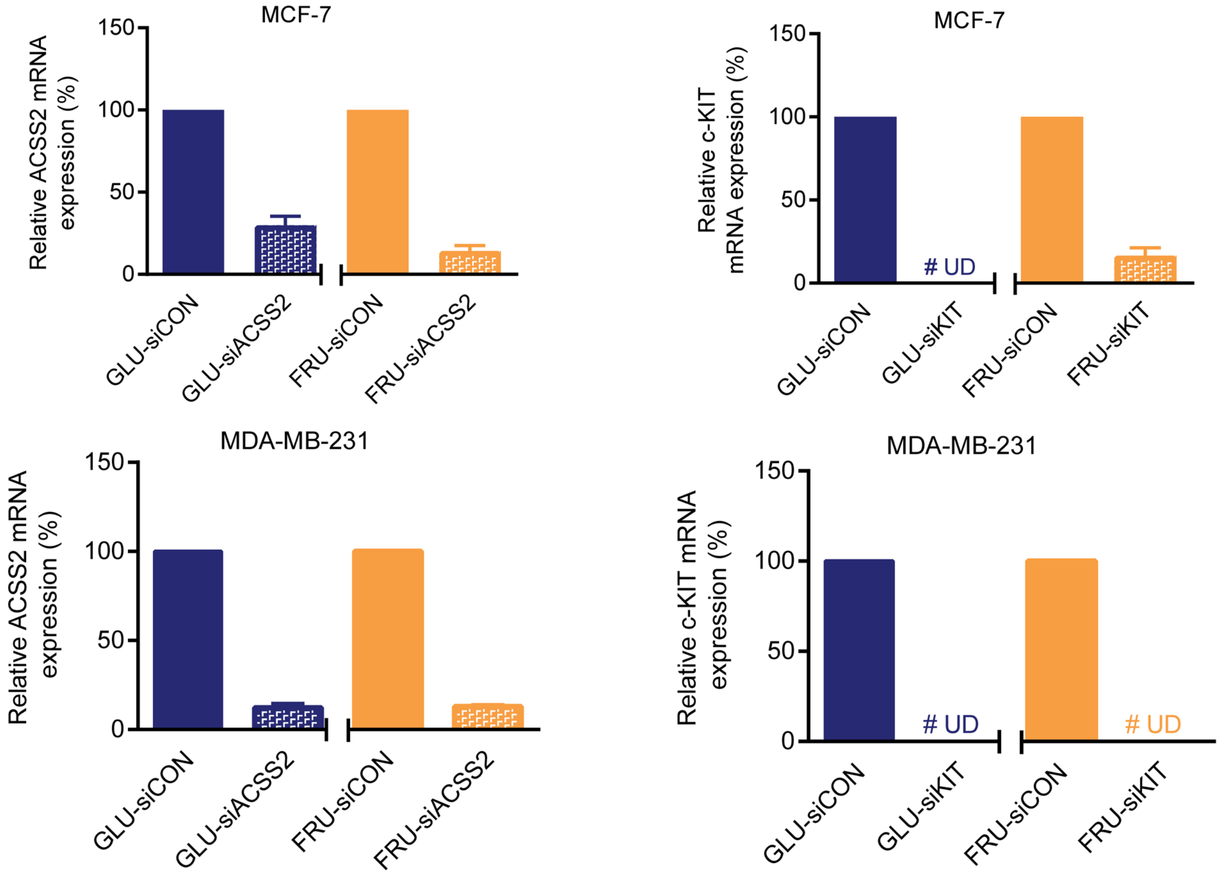

Supplementary Figure 6: Validation of ACSS2 and c-KIT siRNA. Glucose or fructose-adapted MCF-7 and MDA-MB-231 cells were transfected with the indicated siRNA and analyzed by RT-qPCR 48 h later. Relative mRNA expression was calculated against respective glucose or fructose siCON. Mean ± SEM from 3 biological replicates (each being mean of duplicate qPCRs). UD = c-KIT mRNA below limits of detection.
